# Four-Line C_2_*/CH* Optical Sensor for Chemiluminescence Based Imaging of Flame Stoichiometry

**DOI:** 10.3390/s22155665

**Published:** 2022-07-28

**Authors:** Michael E. Tonarely, Tommy Genova, Anthony J. Morales, Daniel Micka, Darin Knaus, Kareem A. Ahmed

**Affiliations:** 1 Department of Mechanical and Aerospace Engineering, University of Central Florida, Orlando, FL 32816, USA; tonarelym@knights.ucf.edu (M.E.T.); tgenovajr@gmail.com (T.G.J.); anthony.morales@ucf.edu (A.J.M.); 2 Creare LLC, Hanover, NH 03755, USA; djm@creare.com (D.M.); dak@creare.com (D.K.)

**Keywords:** species imaging, chemiluminescence, equivalence ratio, combustion, image processing

## Abstract

In the present work, an optical sensor was developed and calibrated for the purpose of non-intrusive equivalence ratio measurements in combustion systems. The sensor incorporates a unique four-line, single-sensor chemiluminescence imaging-based approach, which relies on the ratio of C_2_* and CH* radical-species intensities to obtain measurements of equivalence ratios. The advantage of the four-line sensor is the use of additional filtering to mitigate broadband luminescence signals, and its improvements over conventional two-line chemiluminescence diagnostics are discussed. The sensor was calibrated using a premixed bluff-body jet burner with a propane–air flame operating over a wide range of equivalence ratios. The results showed that the four-line processing technique improved the signal-to-noise ratio of the chemiluminescence images for all test cases. Calibrations of C_2_*/CH* intensity ratio to equivalence ratio were developed for both the four-line and two-line techniques. The calibrations were then used to create maps of local equivalence ratios in the flame-holding region. The maps revealed a non-uniform field of equivalence ratios due to the nature of the radical-species intensity profiles within the flame. Therefore, special consideration is required for calibration in order to accurately quantify equivalence ratios and apply these to diffusion flames.

## 1. Introduction

Turbulent combustion is the predominant mode of converting chemical energy into thermal and mechanical energy for aerospace propulsion, power generation, and transportation. For all applications, the equivalence ratio *Φ* is arguably the most important factor that controls the systems performance [1]. Defined as *Φ* = (*F*/*A*)*/*(*F*/*A*)_stoich_, (*F*/*A*) is the fuel/air mass flow ratio and (*F*/*A*)_stoich_ is the corresponding stoichiometric mass flow ratio value. In particular, local equivalence-ratio measurements would be highly beneficial in diffusion combustors, where flames are stabilized in turbulent flow fields of nonuniform fuel/oxidizer mixture composition. Knowledge of the equivalence ratio would promote a better understanding of combustion heat release, flame stabilization dynamics, and the formation of pollutant emissions such as CO and NO_x_ [2,3,4]. However, measuring local equivalence ratios is challenging due to the high temperature and pressure environment within practical combustors. For this reason, there remains a strong desire to develop non-intrusive sensors that can accurately measure the local equivalence ratio in complex reacting flow fields [5].

One approach to providing non-intrusive and quantitative flame measurements is diagnostic techniques based on the analysis of the flame’s emission spectrum. Among the most popular diagnostic techniques is chemiluminescence, which relies on the imaging of intermediate (or excited) species generated during the chemical reaction. More specifically, excited species such as CH*, C_2_*, or OH* are formed during the reaction of intermediate combustion products or by collision with other bodies [6,7,8]. When the excited species returns to an equilibrium ground-state, it emits photons at a characteristic wavelength or a band of wavelengths [9,10]. Since the emitted light comes from chemical reactions, chemiluminescence is commonly used to identify the structure and location of the flame front, quantify heat release fluctuations, and provide details of the equivalence ratio. Although certain excited species, such as the ones listed above, occur at very narrow bands of wavelengths, there are also radical species that emit light across a wide span of the light spectrum. For hydrocarbon flames, the primary species contributing to broadband chemiluminescence are CO_2_* and CO*, occurring between 300 and 600 nm [9,11,12,13].

A proposed method to quantify the local equivalence ratio from chemiluminescence imaging relies on calculating the intensity ratio of two radical species [11,14,15,16]. Although the intensities of individual radical species are dependent on the equivalence ratio [17,18,19,20], calculating the ratio of two species eliminates some variabilities from the geometric setup or optical instrumentation [21,22,23,24]. Additionally, the ratio of two species has been described to provide more reliable results for turbulent flames which experience local heat-release fluctuations. The selection of the best two species is a function of the flame’s emission spectrum for the given combustor configuration and operating conditions. However, it is beneficial to select species that produce a monotonic relationship between the species intensity ratio and the equivalence ratio. For instance, the intensity ratio of C_2_*/CH* has been shown to produce a monotonic relationship with *Φ* between 0.6 and 1.4 for various hydrocarbon–air flames [11,25,26,27]. The developed calibration curves of C_2_*/CH* vs. *Φ* can then be used to track local or transient flame dynamics, such as heat release-rate fluctuations or lean blowout [28,29]. OH* and CH* are also commonly used pairs of radical species for chemiluminescence imaging [16,25,30,31,32,33]. Chemiluminescence intensity ratios have been used to examine local variations in the species’ intensities and how they relate to the flame structure [15,16,31,32].

Despite the advantages of imaging the species intensity ratio to quantify *Φ*, there remain limitations to the methodology. For instance, the emission intensity from a flame will be dependent on the combustor configuration and operating conditions, as well as the optical setup. Therefore, the calibration and implementation of the technique must be done with care to accurately quantify the equivalence ratio. From an instrumentation standpoint, imaging several radical species would require multiple experimental trials [16,34,35,36] or a multi-camera setup. However, it is preferrable that the light emitted be collected onto a single image sensor to eliminate any errors from misaligned fields-of-view or distortion that could occur with multiple camera/lens setups [37]. Various methodologies have been proposed to collect multiple species images onto a single sensor for CH*, OH*, and C_2_* [12,38]. To minimize the impact of broadband luminescence, an optimized method has been proposed by Kamal [15], that incorporates the subtraction of the broadband signal before calculating the species intensity ratio of CH* to CO_2_*. The method (called the “two-line” method) utilizes a single sensor and was reported to provide reliable equivalence-ratio maps for lean turbulent premixed and stratified flames. A separate study attempted to plot local oxygen/fuel equivalence ratios of diffusion flames using ratios of CH*/CO2* and OH*/CH* [16]. Both methods showed large variation in local equivalence ratios and both correlations failed to reliably match the test-condition value.

The objective of the current work was to explore a novel method to quantify the equivalence ratio from a C_2_*/CH* imaging sensor. The sensor incorporates a “four-line” imaging approach, where four wavelength bands are collected onto a single image sensor. This allows for the broadband signal to be removed from the C_2_* and CH* images before calculating the intensity ratio. The sensor was calibrated using a premixed bluff-body jet flame across a range of equivalence ratios to assess the applicability and limitations of the technique. Additionally, the four-line method explored here is compared to a traditional two-line technique studied in literature. The results show that the four-line technique provides improved signal-to-noise ratios and more qualitatively accurate intensity ratio measurements than the two-line method. The limitations and applicability of the sensor to diffusion flames are also discussed. 

## 2. Materials and Methods

### 2.1. Sensor Design

**Figure 1:**
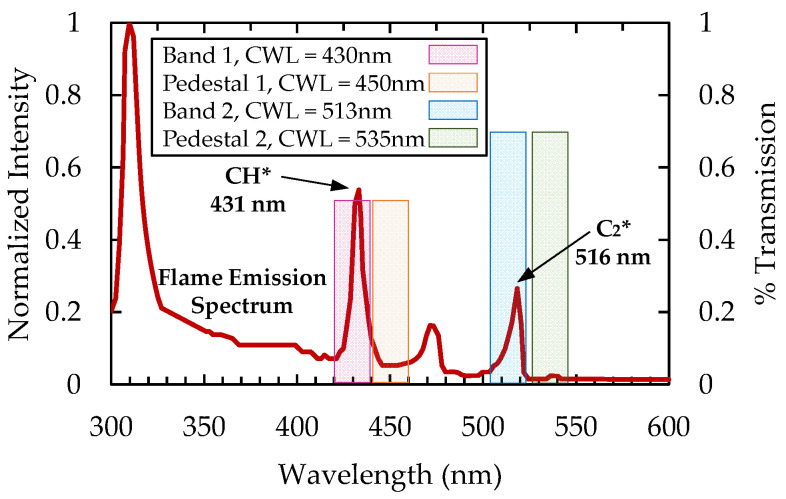
Example of an emission spectrum from a propane–air flame with highlighted regions for optical filtering.

The process of four-line imaging requires two filters to capture the luminosity at desired excited species wavelengths and an additional two filters to capture the broadband luminescence. To help visualize the four-line imaging method, an example of a propane–air flame emission spectrum is shown in Figure 1. There are large peaks of intensities at 431 nm (CH*) and at 516 nm (C_2_*), with normalized intensity values of approximately 0.5 and 0.3, respectively. However, there are also measurable intensities at neighboring wavelengths. For example, in the region near the CH* peak there is broadband intensity with normalized values as large as 0.1. This broadband luminescence is naturally captured in the imaging of the specified radical species. In this case, the broadband intensity can be responsible for as much as 20% of the recorded signal intensity. Reducing the impact of the broadband luminescence intensity can improve the accuracy and applicability of a chemiluminescence-based sensor, thus motivating a four-line imaging approach.

The principle of the four-line technique involves subtracting the broadband luminosity from the radical-species signal. This can be represented by the following equations for CH* [35]:

I_430nm_ = I_CH*_ + I_CO2*,430nm_ (1)

I _CO2*,430_ ≈ I _CO2*,450nm_ (2)

I_CH*_ ≈ I_430nm_ − I_450nm_ (3)

Similar equations can be written for the C_2_* signal by substituting 430 nm and 450 nm with 513 nm and 535 nm, respectively. Subtracting specific wavelength intensities is different than a background subtraction where sensor noise or ambient signal typically caused by light sources or reflections is removed from data sets. Despite the simplicity of the concept, implementing the four-line technique creates additional diagnostic challenges, requiring more cameras or complex optical alignment. A single sensor capable of this diagnostic is desirable for ease of implementation and was developed for the purpose of this study. 

**Figure 2:**
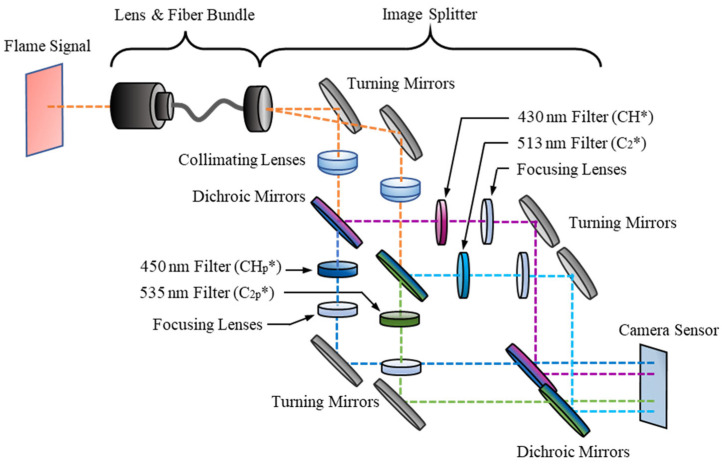
Schematic representation of optical components for four-line chemiluminescence imaging.

A schematic of the optical sensor is displayed in Figure 2 and is comprised of three primary sections: the lens and fiber optic bundle, the image splitter, and the intensified high-speed camera. The first section contains a C-mount lens attached at the distal end of a Schott wound fiber bundle and is used to pass an image of a flame to the image splitter. The fiber bundle is 1.83 m long and it has a resolution of 504 × 504 pixels. 

At its proximal end, the fiber bundle is connected to an optical splitter, where the captured signal is split into four spectral band images using an array of optical elements. Examining the paths of the dashed lines in Figure 2, the flame signal is duplicated using turning mirrors and the first set of dichroic mirrors. Bandpass filters are used for each image to visualize the signal from specific wavelengths. For CH*, the signal is passed through a 430 ± 5 nm filter and the pedestal image through a 450 ± 5 nm filter. Both CH* bandpass filters have a transmissibility of 50%. For C_2_*, the signal passes through a 513 ± 5 nm filter and the pedestal signal through a 535 ± 5 nm filter, each with a transmissibility of 70%. For this study, the ratio of the CH* apertures’ diameters to the C_2_* apertures’ diameters was 2 to 1. The final stage of the image splitter is a pair of turning mirrors and dichroic mirrors which align the four filtered images to create a 2 × 2 array of images which is then passed to the camera sensor. 

The final section of the diagnostic setup is the high-speed imaging camera, a HiCAM Fluo. This camera has a maximum framerate of 1000 fps at its full resolution of 1280 × 1024 pixels. The camera includes an image intensifier that uses a GaAsP photocathode and a P46 phosphor. 

### 2.2. Experimental Facility and Method

**Figure 3:**
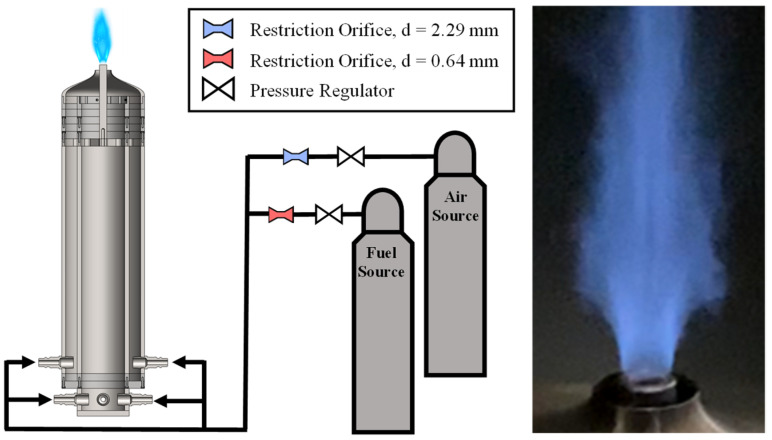
Premixed bluff-body jet burner experiment setup (**left**) and sample flame image (**right**).

This experiment was performed on a bluff-body jet burner, which is shown in the left image of Figure 3. The fuel and air flows are supplied by high-pressure tanks, which are each controlled by a pressure regulator in conjunction with a Dwyer pressure transducer and an O’Keefe precision orifice. The transducers for both flows had max pressure ratings of 300 psi with an error of ±1.5 psi. The air and fuel precision orifices had diameters of 2.29 mm and 0.635 mm, respectively. Following the choke orifices, the two gases are mixed in a series of intersecting hoses to ensure gas premixing before entering the burner through two rings of injectors. Each ring leads to an annular flow channel, and the two flows combine in a converging nozzle just before the facility exit. The exit to the facility is an annulus with an inner diameter of 12.7 mm and an outer diameter of 22.2 mm. 

Propane–air mixtures were tested in a range of equivalence ratios from 0.7 to 1.3 in increments of 0.15 for a total of five test cases, which are summarized in Table 1. Table 1 also includes the Reynolds number, defined as *Re* = *V_e_D_i_*/*ν* where *V_e_* is the exit velocity, *D_i_* is the inner diameter, and *ν* is the kinematic viscosity. Due to the accuracy of the pressure transducers, there was a maximum uncertainty in the fuel and air flow rates of about ±0.005 g/s and ±0.1 g/s, respectively. This propagates to a maximum error in the equivalence ratio of 2.8%.

### 2.3. Data Processing

**Figure 4:**
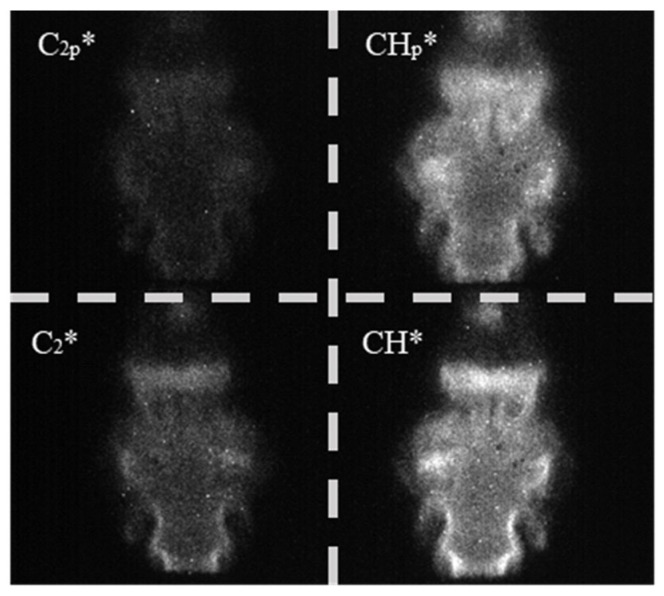
Sample instantaneous image from the four-line chemiluminescence sensor. For clarity, the four individually filtered bands have been segmented.

For the current study, the flames were recorded at a rate of 200 FPS with an exposure time of 250 μs. Each test case was recorded for 5 s to collect 1000 frames for processing, and an instantaneous sample image from the sensor is displayed in Figure 4. The lower half of the image contains the peak wavelength filters, and the upper half the pedestal filters. The first processing step was to average the recorded frames and split the complete sensor image into individual views, resulting in four averaged images with a resolution of 486 × 486 pixels. An additional step was required for mirroring the CH* images, which have been flipped horizontally due to the optics within the sensor.

**Figure 5:**
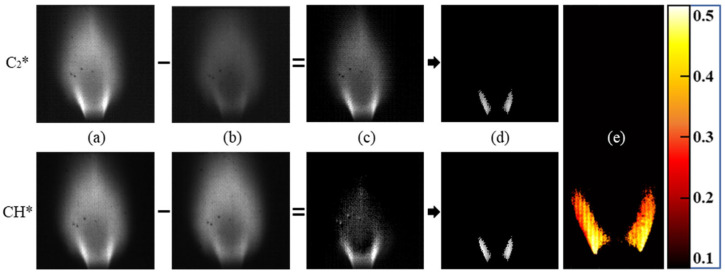
Four-line image processing method. (**a**) Average species signal, (**b**) average broadband signal, (**c**) subtracted signals, (**d**) thresholded flame structure, and (**e**) C_2_*/CH* intensity ratio.

The four-line processing method is displayed in Figure 5. The pedestal signals were subtracted from the peak signal images to isolate the chemiluminescence signals of C_2_* and CH* from any broadband luminescence captured in the peak filters, resulting in the images of Figure 5c. A threshold was applied to the subtracted images at 50% of the maximum signal intensity to focus on the flame structure near the flame holder, which will be discussed further in Section 3.1. The division of the thresholded C_2_* and CH* contours resulted in a plot of C_2_*/CH* intensity ratio, shown in Figure 5e. The mean value and standard deviation for the C_2_*/CH* intensity ratio in the thresholded region were calculated at each equivalence ratio tested, and these were plotted to obtain the calibration of intensity ratio to equivalence ratio. To understand the benefit of including the pedestal filters in the system, a two-line processing method was also utilized. This simplified method only utilized the peak filters for C_2_* and CH* without a signal subtraction, taking the images in Figure 5a and thresholding them to obtain similar structures to the flames shown in Figure 5d to calculate the intensity ratio.

## 3. Results

The following sections compare the results of the four-line and two-line processing techniques on the calibration of the optical sensor and the mapping of equivalence ratios for the premixed propane–air flames. The individual radical-species images were also investigated in order to gain a better understanding of the sensor readings and their potential applicability to transient behavior or diffusion flames.

### 3.1. Signal-to-Noise Ratio (SNR)

**Figure 6:**
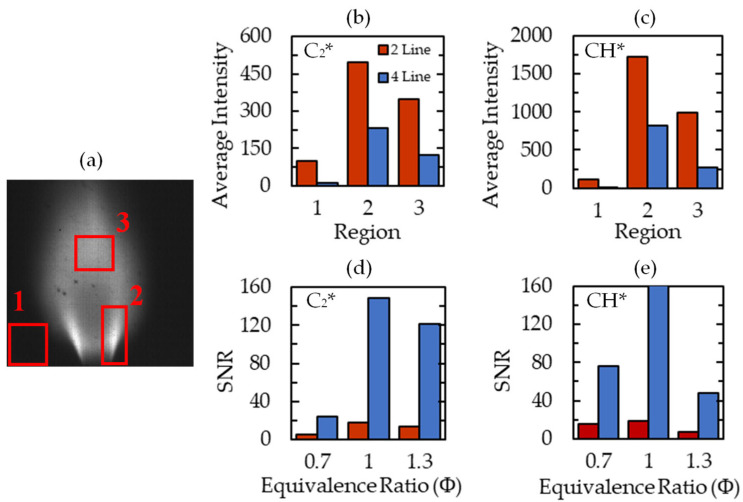
Average signal intensities and signal-to-noise ratio. (**a**) Average C_2_* signal for *Φ* = 0.7 case with labeled regions of interest, (**b**) average C_2_* intensity in ROI, (**c**) average CH* intensity in ROI, (**d**) C_2_* signal-to-noise ratio for *Φ* = 0.7, 1.0, and 1.3 cases, and (**e**) CH* signal-to-noise ratio.

The two-line and four-line image quality are compared in Figure 6. Figure 6a displays an example of the average C_2_* signal, with three regions of interest highlighted. The first region is comprised of the dark background which only contains image noise. The second region contains the high luminosity flame region near the burner lip. The third region captures the downstream burning region. The average intensities of C_2_* and CH* are compared in these regions in Figure 6b,c, respectively, to compare signal clarity between the four-line and two-line processes. 

One benefit of the four-line technique is the reduction of noise in Region 1 due to the subtraction of the pedestal image from the species signal images. The decrease in intensity from the four-line subtraction is also present in Regions 2 and 3. This reduction numerically represents the removal of the broadband luminescence that is captured in the C_2_* and CH* peak filters. There is a greater proportional signal decrease in Region 3 signifying a larger portion of broadband luminescence in that region. For this reason, the primary region of interest for the calibration and mapping of the flame is Region 2, similar to previous studies [15,35]. 

To quantify the differences between the two techniques, the signal-to-noise ratio (SNR) of the images was calculated. This is defined as the ratio of the average intensity in Region 2 to that of Region 1. The SNR dramatically increased in the four-line method for each test case, signifying much greater clarity of the mean flame brush by removing the effect of camera noise. SNR was greatest at *Φ* = 1 because the individual species intensities are largest at this equivalence ratio. Overall, the increased SNR and the reduced average intensities for the four-line processing method are indicative of more quantitative measurement of radical-species intensity.

### 3.2. Calibration of C_2_*/CH* Intensity Ratio to Equivalence Ratio

**Figure 7:**
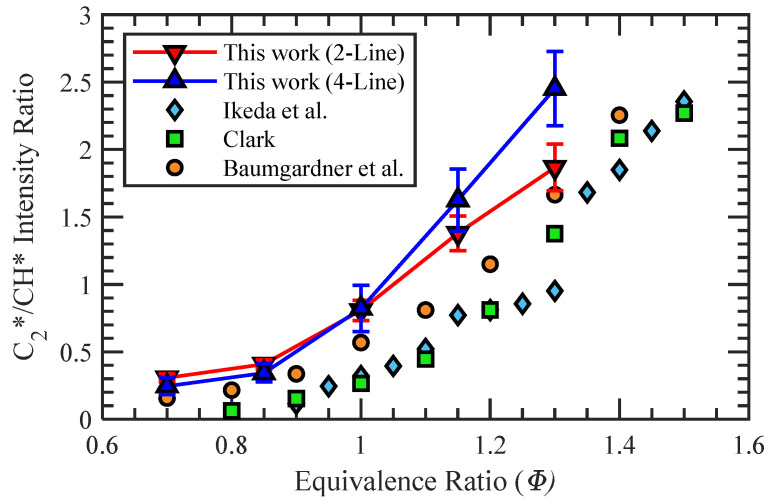
Four-line and two-line calibrations of C2*/CH* intensity ratio to *Φ* for propane–air mixtures.

Utilizing the thresholded flame structure of Region 2, similar to the one shown in Figure 5d, the average C_2_*/CH* intensity value could be obtained at each test condition. The resulting calibrations for the two-line and four-line processes of the premixed propane–air flames are plotted in Figure 7. The primary difference between the two calibrations was the divergence at *Φ* > 1, where the four-line calibration began to increase more rapidly. Additionally, the error bars at each *Φ* represent ±1 standard deviation from the mean C_2_*/CH* value. For the two-line method, the ratio of standard deviation to the mean value at each *Φ* was nominally constant at about 9.3%. However, the standard deviation of the four-line calibration was greater at low *Φ* but decreased with increasing equivalence ratio and had a minimum value of 11.2% at *Φ* = 1.3. Both the four-line and two-line calibrations were monotonic in the tested range of equivalence ratios, which allows for a wide range of applicability of the calibrations for *Φ* mapping. 

The calibration curves in this work show similar trends to those completed in previous studies, which are also presented for comparison [11,26,27]. Variations in the calibrations between this work and other studies are a result of the different diagnostic techniques used in each. The monochromators or spectrometers captured different light emission spectra, and an additional consideration for the calibrations is the removal of any broadband luminescence. In the work by Clark, a single monochromator reading of CO* at 412 nm was used to normalize the readings for OH*, CH*, and C_2_* radiation [11]. The subtraction of the current work is similar to that by Clark in that measurements of broadband radiation are taken at specific wavelengths, but it is done through optical filters rather than with the use of monochromator readings. In the more recent work by Baumgardner et al., a baseline reading of the CO_2_* was acquired and subtracted across the entire spectrum measurement [26]. No mention of a correction for broadband luminescence is made in the work by Ikeda et al. [27]; however, it showed good agreement with Clark’s calibration. Both calibrations created in the present work have intensity ratio values larger than those of previous works over the range of equivalence ratios tested but display similar curvatures signifying agreement in the change of species intensity with *Φ* despite differences in methodology.

The benefit of the calibration curve would be to apply it to a diffusion flame, or flames with nonuniform mixture compositions, and obtain local *Φ*. However, there was already a variation in signal intensity across the premixed mixtures tested in this work. The spatial deviations in C_2_*/CH* are driven by local flame oscillations which will occur for many common flame-holding devices due to the recirculation zone and shear-layer dynamics required to sustain the flame. In the work by Yang et al., there was an even larger deviation in local intensity ratio for the diffusion flame tested which did not match the calibrations of that study [16]. This raises questions regarding the applicability and accuracy for diffusion flames. Thus, we first investigated the applicability of local *Φ* mapping for premixed flames, and quantified spatial variance in *Φ*, which would provide a baseline uncertainty that subsequently propagated onto the diffusion flames.

### 3.3. Local Equivalence-Ratio Mapping

**Figure 8:**
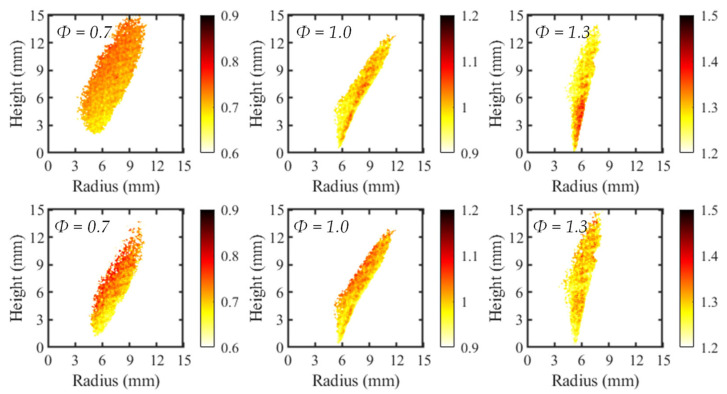
Equivalence ratio maps for two-line (**top**) and four-line (**bottom**) imaging methods.

To investigate the applicability of the local *Φ* mapping, the calibration curves were applied to the average C_2_*/CH* contours. The resulting maps, created using both two-line and four-line calibrations, are presented in Figure 8 for three test cases. Since the flames were axisymmetric, only the right-half of the flame is depicted in the contours. For clarification, the unburned reactants are to the right of the flame and the product region on the left.

Although all flames were premixed, the two-line and four-line processing methods both depicted a variation in the local *Φ*. This was somewhat expected, since there was a small variability in the local intensity ratios used to develop the calibration curves. However, the spatial variance in *Φ* was generally larger for the four-line method due to the greater standard deviation in C_2_*/CH* intensity ratios. 

Additionally, the spatial deviations in *Φ* were not randomly distributed for all test cases. The most common observed trend was a decrease in equivalence ratio with increasing radial position, which was most apparent in the four-line cases for *Φ* = 0.7 and 1, and the two-line case of *Φ* = 0.7. This trend agrees with previous work from Kamal [15], depicting larger values of *Φ* when progressing from reactants to products. This led to the conclusion that equivalence-ratio mapping based on chemiluminescent species intensity ratios is most accurate in high progress variable space. Progress variable space is defined as the position within the flame as it progresses from the reactant side to the product region. In the reactants, the progress variable is equal to zero while in the products it is equal to one. In the current work shown, the progress variable increased from right to left. It was observed for the stoichiometric and rich flames in the two-line process that the highest value of equivalence ratio was found in low progress variable areas. This disagrees with the maps of the four-line method as well as the work by Kamal, suggesting the negative impact of the broadband chemiluminescent signal on the equivalence-ratio mapping technique.

Although the work presented here shows similar trends in progress variable space to the previous work on premixed flames by Kamal, it is currently unclear what caused this result, or if it is relevant for accurate local equivalence-ratio measurements. This prompts an exploration into the cause of the nonuniformity in *Φ* maps. 

### 3.4. Radical-Species Profiles in Progress Variable Space

**Figure 9:**
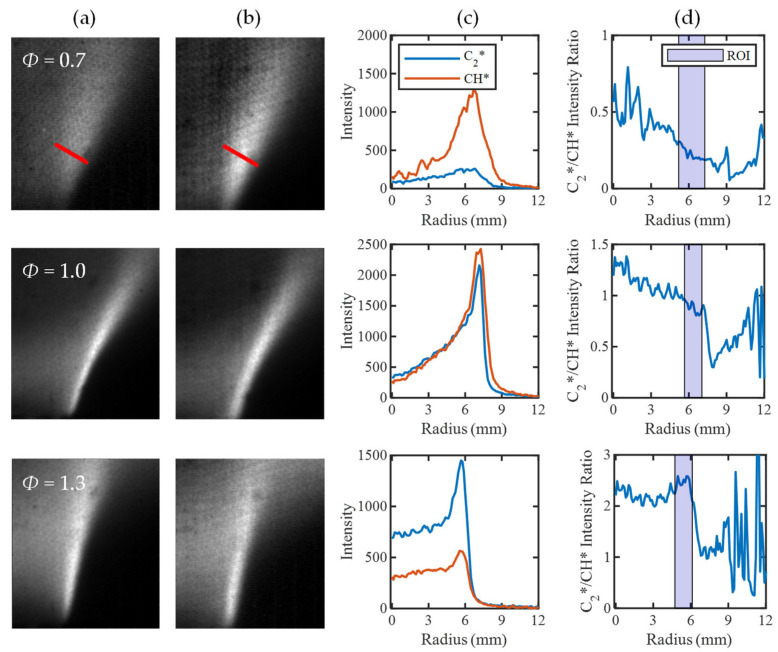
Chemiluminescent decomposition to assess the C2*/CH* intensity ratio in progress variable space, indicated by a red line in the *Φ* = 0.7 case. (**a**) C2* contour, (**b**) CH* contour, (**c**) C2* and CH* intensity profiles, and (**d**) C2*/CH* intensity ratio, ROI highlights thresholded flame region where calibration and mapping are completed.

For the equivalence-ratio maps to increase in value in progress variable space, there must be a variation in the corresponding C_2_*/CH* ratio. Thus, to provide more insight, it is important to understand how the C_2_* and CH* intensities vary across the flame. To do so, mean images of the C_2_* and CH* flame brush from the four-line imaging are first provided in Figure 9a,b. The mean intensities of the two species are then tracked along a line normal to the flame front to replicate the progress variable space shown in Figure 9c. The intensity ratio between the profiles is calculated and presented in Figure 9d, and the highlighted region of interest encompasses the mean flame brush. 

The intensity ratio curves show a similar trend to the corresponding equivalence-ratio maps, in which the intensity ratio increases in progress variable space for the *Φ* = 0.7 and 1 tests. In the case of *Φ* = 1, however, there is a slim region in the low progress variable space where the intensity ratio, and thus the mapped equivalence ratio, increase. This occurs because of an offset between the peaks of C_2_* and CH* across the flame, as C_2_* has a thinner reaction zone than CH* [8,25]. There is a large overlap in the emission regions for both radicals, but the regions that do not coincide will have less defined variation of intensity ratio in progress variable space. For *Φ* = 1.3, there does not appear to be a clear trend in intensity ratio across the flame region. This is due to a greater alignment of the species intensity peaks leading to a more consistent intensity ratio value. Although a constant intensity ratio is expected for premixed flames, the change in intensity ratio value through the flame supports the tailoring of the method to high progress variable space. In this region, the C_2_*/CH* intensity ratio should be unaffected by shifts in the positions of C_2_* and CH* emission peaks in low progress variable space with changes in *Φ*.

## 4. Conclusions

A sensor has been developed which incorporates four species-imaging bands. The four-line imaging technique was found to have improved SNR, resulting in more quantitative species measurements than typical measurement techniques which only utilize two imaging bands. C_2_*/CH* calibrations of both methods have similar values at lean and stoichiometric conditions, while the four-line calibration increases more rapidly in rich test cases. Compared to previous works, the calibrations have similar structure but slightly increased value across all conditions. Maps of local *Φ* were created using both calibrations to investigate the spatial nonuniformity in the tested premixed flames. It was found that there was a trend toward increasing equivalence ratio within the progress variable space. The progression of species production across the reaction zone directly related to the change of intensity ratio in space. Therefore, to apply this technique to diffusion flames, calibration from premixed flames should be completed in the high progress variable space to mitigate the baseline error of local *Φ*.

## Figures and Tables

**Table 1:** Experimental Test Conditions.

*Φ*	${\dot{m}}_{air}\;(g/s)$	${\dot{m}}_{fuel}\;(g/s)$	Exit Velocity (m/s)	Re
0.7	7.5	0.34	25.1	21,005
0.85	7.5	0.41	25.3	21,198
1.0	7.5	0.48	25.5	21,390
1.15	7.5	0.55	25.8	21,583
1.3	7.5	0.62	26.0	21,775

## Data Availability

Not applicable.
